# FedTIP: Communication-Efficient Federated Temporal Prompting for Few-Shot Dynamic Graph Adaptation

**DOI:** 10.3390/e28090989

**Published:** 2026-09-04

**Authors:** Xijun Wu, Xinming Zhang

**Affiliations:** School of Computer Science and Technology, University of Science and Technology of China, Hefei 230027, China; wuxijun@mail.ustc.edu.cn

**Keywords:** federated learning, temporal interaction graphs, communication efficiency, prompt tuning, few-shot learning, dynamic graph adaptation

## Abstract

Temporal interaction streams are often distributed across data owners, while downstream labels and communication budgets remain limited. Existing temporal graph prompts typically adapt at a centralized trainer, whereas federated graph methods commonly exchange larger trainable states. This paper presents FedTIP, a prompt-level federated method for client-local few-shot adaptation of temporal interaction graphs. A centralized historical stage constructs a temporal knowledge bank whose encoder is frozen during downstream federation. Clients optimize interaction-conditioned prompts on local supervised events and transmit a compact prompt-side message. Prototype anchoring and a reliability rule based on support coverage and update magnitude combine heterogeneous client updates, while prompt-side proximal regularization and stage-scoped negative sampling preserve the temporal protocol. Experiments on Wikipedia, Reddit, and MOOC cover temporal node classification and transductive and inductive link prediction. FedTIP records the highest mean AUC-ROC in the nine reported task–dataset cells, with gains of 1.83–14.69 points over the strongest federated baseline in each cell. Its measured cumulative float32 uplink is 0.40 MiB per downstream task, 64.6–99.6% below the evaluated baselines.

## 1. Introduction

Online services generate continuous streams of timestamped interactions among users, content, courses, and other entities. Temporal graph models preserve the order and timing of these events: JODIE and DyRep model evolving node states [1,2], whereas TGAT and TGN use temporal attention or memory-based message passing [3,4]. Their evaluation must also respect what was observable at each time. Surveys and benchmark studies identify chronological splitting, inductive status, and time-valid negative sampling as central concerns [5,6,7]. Thus, adapting a temporal graph model is constrained by both scarce downstream supervision and the history available before each prediction.

In many deployments, organizations retain their own downstream evidence. Federated averaging learns from decentralized data through iterative aggregation of local model updates [8], and federated graph learning extends this access pattern to graph-structured data [9,10]. Communication can be reduced by jointly optimizing model compression and resource allocation [11] or by transmitting sparse gradients from heterogeneous devices [12]. Knowledge distillation offers another compact exchange mechanism for statistically heterogeneous clients [13]. These studies motivate the setting considered here: the client-owned observations form a chronologically evolving interaction stream, only a few labeled events are available for each downstream task, and repeated uplink transmission is constrained.

Pre-training and prompting provide a natural starting point. Prompt tuning was introduced as a parameter-efficient way to adapt a frozen model [14]. Graph and dynamic graph pre-training learn reusable representations from historical structure [15,16,17]; GraphPrompt, GPF, and All-in-One then adapt graph encoders through compact prompts [18,19,20]. Temporal prompt methods introduce time-aware or node–time conditional adaptation [21,22], but their downstream support is normally available to a centralized trainer.

Federated graph learning takes the complementary view that downstream observations remain distributed. Static or snapshot-based approaches exchange predictions or graph model parameters [23,24,25]. Federated dynamic-graph systems exchange temporal–spatial states, dynamic graph parameters, or task-specific temporal representations [26,27,28]; FedLink formulates federated temporal link prediction in a non-archival NeurIPS workshop manuscript [29]. These two lines of research leave a specific adaptation problem unresolved. Temporal prompting generally assumes centralized access to downstream support, whereas federated dynamic-graph methods coordinate substantially larger model or representation state. Simply applying FedAvg to a compact prompt does not resolve the resulting evidence imbalance: a fixed prompt cannot respond to event-specific partners, time intervals, and pre-event states, and a client with only a few chronological support events may observe one class or produce an update with an atypical magnitude. The challenge is therefore not only to reduce trainable parameters, but also to determine how event-conditioned updates and sparse class evidence should influence shared adaptation.

**Figure 1:**
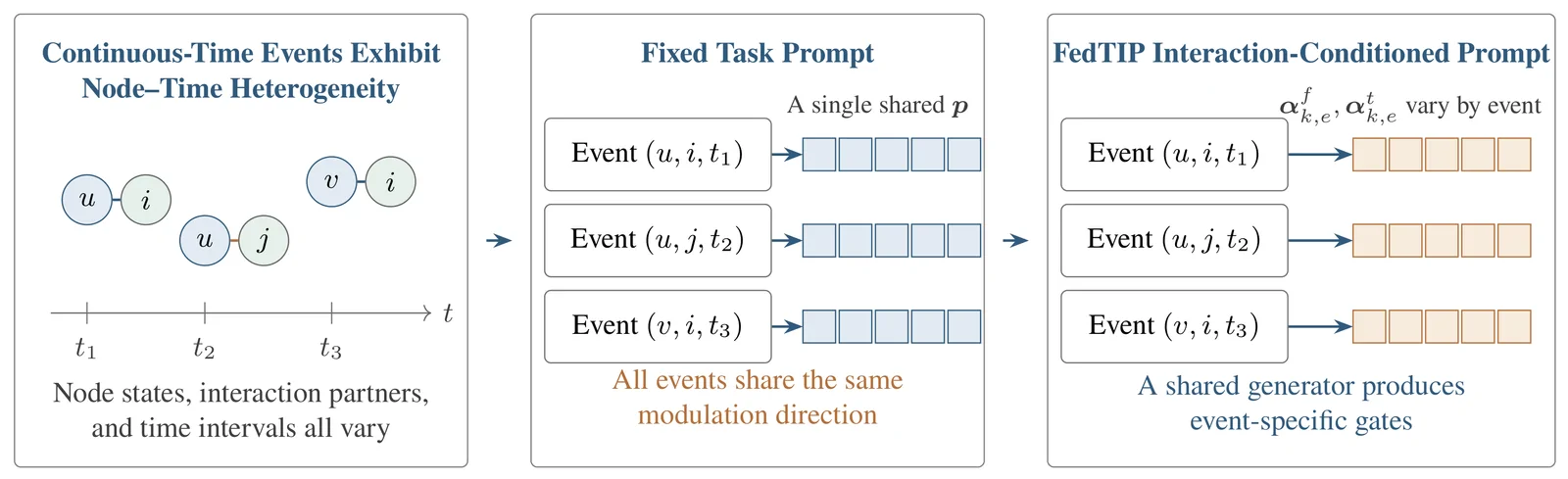
Fixed and interaction-conditioned prompts for client-local temporal adaptation. FedTIP produces event-specific feature and temporal gates from the pre-event interaction context.

Figure 1 illustrates the representation side of this problem. Continuous-time events differ in their pre-event node states, interaction partners, and time intervals, whereas a fixed task prompt applies one modulation direction to every event. An interaction-conditioned adapter can instead derive feature and temporal gates from each observable event context. On the aggregation side, client updates must also be interpreted in light of the sparse and potentially single-class support from which they were estimated.

FedTIP addresses these coupled representation and aggregation problems by federating an interaction-conditioned adapter over a frozen temporal knowledge bank. Its key mechanism is a coverage-aware reliability rule that combines local label coverage with prompt update magnitude and applies the resulting weights consistently to both prompt deltas and class-anchor updates. Consequently, sparse client evidence influences the shared prompt and prototype geometry through the same reliability semantics. The structured uplink further admits a replace-one sensitivity bound for class prototypes and an additive sufficient-statistics decomposition for secure summation, while raw downstream support events remain with their owners.

FedTIP builds on frozen temporal encoding, proximal regularization, prototypical objectives, and federated averaging; its methodological novelty lies in jointly governing event-conditioned prompt updates and class-anchor geometry through coverage-aware reliability under chronological few-shot support.

The main contributions are summarized as follows:

- We formulate chronologically constrained federated temporal prompt adaptation and instantiate it with a frozen temporal knowledge bank and client-side interaction-conditioned adapters. Raw downstream support events remain with their owners, while recurrent federation exchanges structured prompt-side messages rather than the full temporal model.
- We introduce coverage-aware joint prompt–prototype aggregation. A reliability rule combines local label coverage and prompt update magnitude, and the resulting weights govern both prompt deltas and class-anchor contributions, coupling representation adaptation with prototype alignment under sparse and class-imbalanced client support.
- We characterize the structured message interface through a replace-one prototype-sensitivity bound and an additive sufficient-statistics decomposition, establishing compatibility with sensitivity-calibrated prototype release and secure summation.
- We evaluate FedTIP on three temporal interaction datasets and three task families. It obtains the highest reported mean AUC-ROC in all nine same-access cells among the evaluated methods; matched TGAT/TGN comparisons, component ablations, hyperparameter and partition analyses, and communication measurements isolate the behavior and cost of the proposed mechanism.

The remainder of this paper is organized as follows. Section 2 reviews the related work. Section 3 presents the system model and problem formulation. Section 4 describes FedTIP. Section 5 presents the experimental setup, results, and limitations. Section 6 concludes the paper.

## 2. Related Work

### 2.1. Temporal Interaction Modeling and Prompt Adaptation

Dynamic graph representation learning differs in how time is exposed to the encoder. Discrete-time methods transform an event stream into snapshots and apply static or recurrent graph encoders. GCN and GAT provide representative spatial operators [30,31], while ROLAND evolves node embeddings across snapshots [32]. Continuous-time DGNNs operate closer to the interaction stream. JODIE and DyRep model coupled trajectories or point-process dynamics [1,2]. TGAT and TGN use temporal attention or memory states [3,4], while TREND and CAW capture event-node dynamics or causal walks [33,34]. Surveys establish the need for chronological and inductive evaluation [5]; benchmark studies specify time-valid splits and negative-sampling protocols [6,7].

Pre-training reduces dependence on downstream labels by learning reusable graph representations. DDGCL and CPDG exploit temporal regularities in historical streams [16,17]. Prompt learning changes the unit of adaptation: GraphPrompt, GPF, and All-in-One use compact graph prompts [18,19,20], and surveys organize these methods as parameter-efficient graph adaptation [35,36]. Temporal prompting introduces time-aware or node–time conditional prompts [21,22].

### 2.2. Communication-Efficient Federated Adaptation

FedAvg establishes the iterative model-averaging baseline for decentralized learning [8]. Communication-aware variants reduce transmitted states through model compression [11], sparse gradients [12], or distillation [13].

Statistical heterogeneity motivates changes to aggregation. FedProx constrains local drift, SCAFFOLD reduces client update variance, and FedNova corrects objective inconsistency under unequal local computation [37,38,39]. FedDyn adds dynamic regularization, while FedBN retains local normalization statistics under feature shift [40,41]. FedProto establishes a prototype-level federation pattern in which clients upload class prototypes, the server aggregates them, and global prototypes return to clients [42]. FedHide subsequently characterizes leakage from shared class prototypes and replaces true prototypes with proxy representations [43]. Differentially private prototype learning further shows how a frozen public encoder and private class representations can be coupled through sensitivity-calibrated prototype release [44]. Ditto relates regularized local adaptation to personalized federation [45].

### 2.3. Federated Graph and Dynamic Graph Learning

Federated graph learning makes graph ownership and partitioning explicit. Surveys organize the field around data privacy, graph heterogeneity, and structured collaboration [9,10]. FedGNN addresses privacy-preserving recommendations [23], FedSage+ generates missing cross-client neighbors [24], and FedGCN studies communication–convergence trade-offs for graph convolution [25]. FedGTA uses topology-aware aggregation [46]; FedGraphNN and FedGraph provide benchmark infrastructure [47,48].

Federated dynamic graph learning provides the nearest temporal baseline family. Feddy and STFL study dynamic GNN federation or temporal–spatial graph learning [26,27]. FedDGL models heterogeneous temporal evolution [28], and FedLink addresses federated dynamic link prediction [29].

Taken together, prior studies address temporal prompting, communication-efficient federation, prototype aggregation, and federated dynamic graph learning largely as separate problems. They do not jointly model event-conditioned prompt adaptation and class-prototype aggregation when chronological supervision is both client-owned and extremely sparse.

## 3. System Model and Problem Formulation

FedTIP considers a coordinating server and independently owned clients under a four-part access model. Historical interactions are available centrally for pre-training; sampled downstream support events are client-owned and used for local adaptation; a global downstream validation segment is visible to the server for model selection; and a global test segment is visible to the server only for final scoring. The locality scope covers the few-shot downstream support sets within this centralized pre-training and evaluation protocol. The objective is to adapt a temporal interaction model while keeping those support events with their owners, respecting chronological visibility, and measuring cumulative uplink payload.

### 3.1. Temporal Interaction Graph

Let G=(V,E) be a temporal interaction graph represented as a chronologically ordered event stream

(1) $$E={\left\{e_{n}=\left(u_{n},i_{n},t_{n},y_{n},x_{n}\right)\right\}}_{n=1}^{N},\;t_{1}\leq t_{2}\leq \cdots \leq t_{N}.$$

Here $u_{n}$ is a source-side node, $i_{n}$ is a destination-side node, $t_{n}$ is the interaction timestamp, $y_{n}$ is an optional dynamic state label associated with the event or source node, and $x_{n}\in R^{d_{e}}$ is an edge feature vector. For any time *t*, $H^{t^{-}}$ denotes the events observable strictly before *t*. Before training, the manifest assigns every raw event to one of six mutually exclusive outcomes: historical pre-training, pre-training validation, an observed downstream supervised pool $E_{\sup}$, an inductive-node exclusion from the downstream training candidate window, downstream validation $E_{\mathrm{val}}$, or downstream test $E_{\mathrm{test}}$. The inductive test set is a flagged subset of $E_{\mathrm{test}}$ involving a typed user or item node removed from $E_{\sup}$. Earlier client updates and training negatives draw exclusively from information available before validation and test. The global downstream validation segment selects method-specific hyperparameters and is substantially larger than the 30-event support set; this stronger server-visible resource is part of the reported protocol.

### 3.2. Deployment and Communication Model

The server coordinates *K* clients and maintains the shared model state. Before the first downstream task, it deploys the frozen temporal bank to participating clients. In round *r* of task τ, it broadcasts the current adapter and anchor prototypes and receives the FedTIP uplink message   

(2) $$M_{k}^{\left(r\right)}=\left(\Delta \Omega_{k}^{\left(r\right)},{\left\{q_{k,c}^{\left(r\right)},n_{k,c}\right\}}_{c\in Y_{k}},b_{k},{∥\Delta \Omega_{k}^{\left(r\right)}∥}_{2}\right).$$

The first component is the prompt-adapter delta; the remaining components are class prototype means and counts, a label-coverage score, and the update norm. This tuple constitutes the FedTIP uplink message. Raw downstream support events remain with their assigned clients, while the centrally constructed frozen temporal encoder is deployed separately before downstream adaptation.

The system abstraction assumes that every selected active client completes its local computation and upload within a round. Communication accounting sums float32 values transmitted from clients to the server over all active clients and rounds for one downstream task. Cumulative uplink payload provides the common cross-method measurement, while Section 5.4 analyzes initialization and recurrent downlink from each method’s exchanged objects and communication cadence.

### 3.3. Federated Few-Shot Adaptation

There are *K* clients C={1,…,K}. In the main user-iid profile, the split manifest first fixes a user-ownership map $\pi_{U}:V_{U}\to C$ and then assigns every downstream event e=(u,i,t,y,x) to $\pi \left(e\right)=\pi_{U}\left(u\right)$. Consequently, all events of one user have exactly one client owner. Client *k* owns a supervised pool $E_{\sup,k}$, and the client pools form a disjoint union of $E_{\sup}$. Label-skewed and activity-skewed profiles change the user-to-client map but retain the same pre-training-time materialization and method-independent ownership rule. For a downstream task τ, an active client set $A_{\tau }\subseteq C$ is selected from the manifest. Each active client receives a small local support set

(3) $$D_{\tau ,k}\subseteq E_{\sup,k}.$$

The support rows and their labels remain inside the assigned client boundary. The manifest also assigns validation and test rows to their user owners; the disjoint client views form the corresponding global segments, whereas model selection and final scoring use their unions at the server. This definition is method-agnostic: a baseline may adapt a prediction head, a prompt, or a larger model state, but every method follows the same support ownership, active-client set, chronological split, and common server-side evaluation segments.

### 3.4. Evaluation Tasks

The evaluation contains three task families. Temporal node classification predicts a state label for the source-side node at the interaction time. Transductive link prediction evaluates later interactions among nodes observed before the downstream test stage. Inductive link prediction restricts scoring to test events containing at least one typed node excluded from the downstream supervised pool. Each positive link example is paired with one negative destination. A negative event (u,j,t) is valid only if the interaction is absent at time *t* under the stage-specific history allowed by the protocol. All methods use the same deterministic stage-valid sampling rule and task/split seed. We materialize this rule for all 9000 task-support events, and Section 5.1 reports the resulting collision checks.

### 3.5. Trust and Privacy Boundary

Attack-oriented multimedia-security studies evaluate exposed intermediate structures under explicit adversarial capabilities; for example, video cryptanalysis considers chosen-plaintext, known-plaintext, and chosen-ciphertext attacks against internal encryption transformations [49]. Following this principle, FedTIP’s threat model specifies message visibility and manipulation surfaces for prompt and prototype disclosures.

The protocol assumes that the coordinator and clients execute the prescribed optimization and reporting rules. The confidentiality analysis treats the coordinator as honest-but-curious after it receives the structured uplink messages, and the integrity analysis treats prompt deltas, prototype summaries, counts, coverage values, and update norms as possible manipulation surfaces. Raw downstream support events remain with their manifest-assigned owners. The coordinator observes $M_{k}^{\left(r\right)}$; a participating client observes the frozen bank, the current adapter, and server-aggregated anchor prototypes. Client-specific prototypes are used to construct the global anchors and are not redistributed to other clients. The bank broadcast defines the reverse disclosure path because its centrally derived historical statistics and negative-sampling support are visible to participating clients.

The prototype message admits a direct sensitivity analysis. Let the representations used in class *c* be clipped so that ${∥{\tilde{h}}_{k,e}∥}_{2}\leq C_{\mathrm{emb}}$, and consider fixed-size replace-one adjacency for $D_{k,c}$. Since   

(4) $$q_{k,c}=\frac{1}{n_{k,c}}\sum_{e\in D_{k,c}}{\tilde{h}}_{k,e},$$

its $l_{2}$-sensitivity is bounded by   

(5) $$\Delta_{2}\left(q_{k,c}\right)\leq \frac{2C_{\mathrm{emb}}}{n_{k,c}}.$$

The bound decreases with the local class count. For $n_{k,c}=1$, the prototype equals the clipped representation of that event, making the small-count case the most informative message. This result formalizes the prototype exposure identified in prototype-sharing studies [43]. Across the 500 task–client pairs per dataset, the realized supports form 604, 602, and 625 local class prototypes on Wikipedia, Reddit, and MOOC. Of these, 104 (17.22%), 102 (16.94%), and 118 (18.88%) are single-event prototypes. These counts characterize how often the sensitivity bound operates in its highest-exposure small-support regime.

FedTIP’s weighted prompt and anchor updates can be expressed through four additive sufficient statistics:

(6) $$\begin{array}{cccc} S_{\Omega } & =\sum_{k\in A_{\tau }}n_{k}r_{k}\Delta \Omega_{k},\; & Z_{\Omega } & =\sum_{k\in A_{\tau }}n_{k}r_{k},\\ S_{c} & =\sum_{k:c\in Y_{k}}n_{k,c}r_{k}q_{k,c},\; & Z_{c} & =\sum_{k:c\in Y_{k}}n_{k,c}r_{k}. \end{array}$$

The server updates use $S_{\Omega }/Z_{\Omega }$ and $S_{c}/Z_{c}$; therefore, the aggregation rule is compatible with secure summation of the numerators and denominators [50] without changing the client objective or prompt parameterization. In the main profile, these statistics combine the five active clients selected for each task, and clients receive the resulting global adapter and anchors. Equation (5) also gives a differential-privacy-compatible prototype map

(7) $${\tilde{q}}_{k,c}=q_{k,c}+N\;\left(0,\sigma^{2}\Delta_{2}^{2}I\right),$$

where $\Delta_{2}=2C_{\mathrm{emb}}/n_{k,c}$ links the perturbation scale to the proved prototype sensitivity. This construction connects the FedTIP message interface to established differential-privacy mechanisms and private prototype learning [44,51].

## 4. FedTIP Method

FedTIP federates an interaction-conditioned prompt adapter while keeping the temporal encoder frozen. Its central mechanism is coverage-aware joint prompt–prototype aggregation: reliability derived from local label coverage and update magnitude weights both prompt deltas and class-anchor contributions. A temporal knowledge bank supplies historical representations, while the shared reliability semantics govern how heterogeneous few-shot evidence changes the global adapter and prototype geometry.

### 4.1. Overview and Federated Visibility

**Figure 2:**
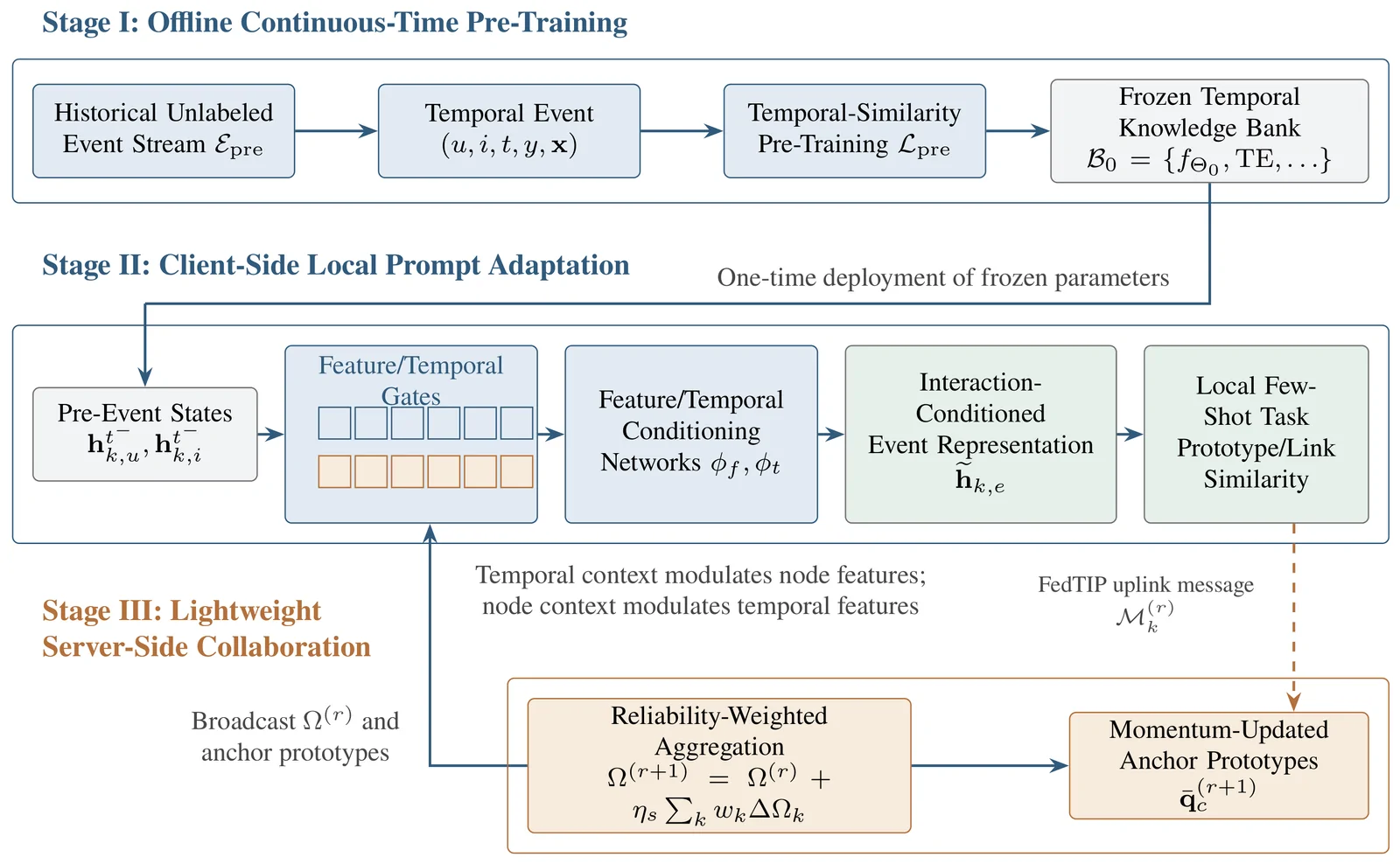
FedTIP workflow. Historical pre-training constructs a frozen temporal bank; client-side adaptation updates event-conditioned prompts; the server aggregates FedTIP uplink messages and updates anchor prototypes.

Figure 2 organizes FedTIP into three stages. Stage I constructs the frozen bank $B_{0}$ from chronological historical interactions. In Stage II, parallel feature and temporal branches map each client-visible event context to an interaction-conditioned representation. In Stage III, clients transmit $M_{k}^{\left(r\right)}$ from Section 3; the server updates the adapter and anchor prototypes.

The encoder, temporal memory, neighbor states, and time encodings are frozen after the centralized historical stage. Downstream visibility and communication follow Section 3; repeated client-to-server transmission is confined to $M_{k}^{\left(r\right)}$.

Five elements define the method. The bank $B_{0}$ contains the frozen temporal encoder and historical metadata. The adapter Ω contains the feature gates, temporal gates, and task template. Local prototypes $\left\{q_{k,c}\right\}$ summarize class geometry, while global anchor prototypes are maintained separately at the server. The reliability score $r_{k}$ uses label coverage and update magnitude. The filtered negative index $I_{\mathrm{neg}}$ is a protocol object, separate from the neural modules.

The bank precedes downstream task sampling, local updates use the frozen bank and preceding events in the assigned task support, and server-side adaptation uses only the FedTIP uplink messages.

### 4.2. Temporal Knowledge Bank and Interaction Prompting

The temporal knowledge bank defines the shared information available before downstream labels are sampled. It is broader than a parameter checkpoint because it also contains time encodings, historical statistics, and negative-sampling support for permitted stages. The prompt adapter then specializes this shared temporal knowledge to a client-local downstream event without changing the frozen encoder. Anchor prototypes are initialized separately on the server and evolve only during downstream collaboration.

During historical bank construction, the frozen encoder produces temporal node states

(8) $$h_{u}^{t^{-}},h_{i}^{t^{-}}=f_{\Theta_{0}}\left(u,i,t,H^{t^{-}}\right),$$

where $H^{t^{-}}$ denotes the chronological history before *t* in the pre-training stream. The encoder is trained with temporal link prediction on the historical stream. Given a positive interaction (u,i,t), a negative destination is sampled from the pre-training filtered candidate set

(9) $$N_{t}^{\mathrm{pre}}\left(u\right)=\left\{j\in V_{I}:\left(u,j,t\right)\notin H_{\mathrm{pre}}^{t}\right\},$$

where $V_{I}$ is the destination-side node set and $H_{\mathrm{pre}}^{t}$ contains pre-training interactions observable up to time *t*. The filtering rule prevents a same-time or historical positive interaction from being used as a negative example whenever the protocol can detect it. The pre-training objective is   

(10) $$L_{\mathrm{pre}}=-\log\frac{\exp(s\left(h_{u}^{t^{-}},h_{i}^{t^{-}}\right)/\tau )}{\exp\left(s\left(h_{u}^{t^{-}},h_{i}^{t^{-}}\right)/\tau \right)+\exp\left(s\left(h_{u}^{t^{-}},h_{j}^{t^{-}}\right)/\tau \right)},$$

where $j∼N_{t}^{\mathrm{pre}}\left(u\right)$, s(·,·) is cosine similarity or a bilinear score, and τ is a temperature. The output of this stage is the frozen bank

(11) $$B_{0}=\left\{f_{\Theta_{0}},\mathrm{TE},S_{\mathrm{hist}},I_{\mathrm{neg}}\right\},$$

which contains the encoder, time encoding, historical recency and frequency statistics, and stage-scoped negative-sampling support. The bank provides temporal knowledge to downstream clients without requiring them to learn long-range event dynamics from a handful of labels.

During downstream adaptation, client-side state construction uses the frozen historical bank and the client’s task support observed before the current event. For client *k* in task τ, the event state is written as

(12) $$h_{k,u}^{t^{-}},h_{k,i}^{t^{-}}=f_{\Theta_{0}}\left(u,i,t,H_{\mathrm{pre}}^{t^{-}}\cup H_{\tau ,k}^{t^{-}}\right),$$

where $H_{\mathrm{pre}}^{t^{-}}$ is the centrally constructed bank context and $H_{\tau ,k}^{t^{-}}=\left\{e\in D_{\tau ,k}:t_{e}<t\right\}$ contains preceding events in the manifest-assigned task support. Thus, task-specific context is restricted to the client’s chronologically preceding support, while the frozen historical bank remains shared.

FedTIP adapts the frozen temporal encoder with a temporal interaction prompt. Generating the prompt from each event context allows the same global adapter to react differently to recent, old, high-activity, or sparse task support. For each local event e=(u,i,t,y,x), client *k* constructs

(13) $$z_{k,e}=[h_{k,u}^{t^{-}}∥h_{k,i}^{t^{-}}∥\mathrm{TE}\left(t\right)∥x∥\Delta_{k,u}\left(t\right)∥\Delta_{k,i}\left(t\right)],$$

where TE(t) is the time encoding, $\Delta_{k,u}\left(t\right)$ and $\Delta_{k,i}\left(t\right)$ are source- and destination-side recency features available from the frozen bank and preceding task support, and the frozen states are computed under the access rule above. The adapter then generates two gates:

(14) $$\begin{array}{cc} \alpha_{k,e}^{f} & =\sigma (W_{f}\phi_{f}\left(z_{k,e}\right)+b_{f}), \end{array}$$

(15) $$\begin{array}{cc} \alpha_{k,e}^{t} & =\sigma (W_{t}\phi_{t}\left(z_{k,e}\right)+b_{t}), \end{array}$$

where $\phi_{f}$ and $\phi_{t}$ are bottleneck networks and σ is a sigmoid function. Both branches receive the same event context $z_{k,e}$ and are evaluated in parallel. Consequently, the temporal encoding and recency terms in $z_{k,e}$ can modulate the feature gate, while the pre-event node states can modulate the temporal gate, without either bottleneck network consuming the output of the other. The feature gate controls which structural interaction dimensions should be amplified for the current event, while the temporal gate controls how strongly the time encoding enters the downstream representation. The prompted representation is

(16) $${\tilde{h}}_{k,e}=\psi \left(\alpha_{k,e}^{f}⊙\left[h_{k,u}^{t^{-}}∥h_{k,i}^{t^{-}}\right],\alpha_{k,e}^{t}⊙\mathrm{TE}\left(t\right),x\right).$$

Here $\psi :R^{2d_{h}}\times R^{d_{t}}\times R^{d_{e}}\to R^{d_{p}}$ is a lightweight three-input task template. It concatenates the gated source–destination state, gated time encoding, and edge features and maps them to a $d_{p}$-dimensional representation used by the task loss. Only the adapter

(17) $$\Omega =\{W_{f},W_{t},\phi_{f},\phi_{t},b_{f},b_{t},\psi \}$$

is trainable and communicated.

### 4.3. Downstream Objectives and Temporal Regularization

Downstream learning has two layers. The task loss makes the prompt useful for node classification or link prediction. The temporal and federated regularizers keep the client-local update compatible with the shared temporal geometry when only a few events are labeled. For a fixed downstream task τ, we omit the task index and write $D_{k}$ for $D_{\tau ,k}$.

For temporal node classification, FedTIP uses the prototypical classification objective [52] and forms a class mean from each client’s local support set:

(18) $$q_{k,c}=\frac{1}{|D_{k,c}|}\sum_{e\in D_{k,c}}{\tilde{h}}_{k,e}.$$

Here $D_{k,c}$ denotes the subset of $D_{k}$ whose label is *c*. The same local support events form the prototypes and optimize the support loss below, while validation and test events remain outside this computation. The resulting in-support regularizer can produce optimistic local losses when self-inclusion occurs in a very small class. The local classification loss is

(19) $$L_{k}^{\mathrm{nc}}=-\sum_{\left(e,y\right)\in D_{k}}\log\frac{\exp(s\left({\tilde{h}}_{k,e},q_{k,y}\right)/\tau )}{\sum_{c}\exp\left(s\left({\tilde{h}}_{k,e},q_{k,c}\right)/\tau \right)}.$$

For temporal link prediction, FedTIP uses the same similarity template as pre-training. For any candidate destination *v*, ${\tilde{h}}_{k,u,v,t}$ denotes Equation (16) evaluated with the pre-event states of (u,v) and contains neither the link label nor a post-event state:

(20) $$\begin{array}{cc} L_{k}^{\mathrm{lp}}\; & =-\sum_{\left(u,i,j,t\right)\in D_{k}}\log\frac{\exp(s\left({\tilde{h}}_{k,u,i,t},h_{k,i}^{t^{-}}\right)/\tau )}{Z_{u,i,j,t}}, \end{array}$$

(21) $$\begin{array}{cc} Z_{u,i,j,t}\; & =\exp\left(s\left({\tilde{h}}_{k,u,i,t},h_{k,i}^{t^{-}}\right)/\tau \right)+\exp\left(s\left({\tilde{h}}_{k,u,j,t},h_{k,j}^{t^{-}}\right)/\tau \right). \end{array}$$

The local prototypes can drift sharply when a client receives only a few labeled events. FedTIP therefore maintains server-side anchor prototypes ${\bar{q}}_{c}^{\left(r\right)}$ aggregated from previous rounds. Client *k* regularizes its local prototypes through

(22) $$L_{k}^{\mathrm{anchor}}=\sum_{c\in Y_{k}}\rho_{k,c}\left(1-s(q_{k,c},{\bar{q}}_{c}^{\left(r\right)})\right),$$

where $Y_{k}$ is the set of labels observed by client *k*, $n_{k,c}=\left|D_{k,c}\right|$, $n_{k}=\left|D_{k}\right|$, and $\rho_{k,c}=n_{k,c}/n_{k}$. Thus, classes represented by more local support events contribute proportionally more to the anchor penalty.

After each round, the server updates the anchor prototypes from uploaded summaries while raw downstream support events remain with their assigned clients:

(23) $$\begin{array}{cc} {\hat{q}}_{c}^{\left(r+1\right)}\; & =\frac{\sum_{k:c\in Y_{k}}a_{k,c}q_{k,c}}{\sum_{k:c\in Y_{k}}a_{k,c}+\epsilon }, \end{array}$$

(24) $$\begin{array}{cc} {\bar{q}}_{c}^{\left(r+1\right)}\; & =\mathrm{norm}\left(\left(1-\beta \right){\bar{q}}_{c}^{\left(r\right)}+\beta {\hat{q}}_{c}^{\left(r+1\right)}\right), \end{array}$$

where $a_{k,c}=r_{k}n_{k,c}$ is an unnormalized class-specific anchor weight, β controls anchor momentum, and norm(·) denotes unit normalization.

For downstream link prediction, temporal negatives are sampled from the client-visible candidate set

(25) $$N_{k,t}^{\mathrm{tr}}\left(u\right)=\left\{j\in V_{I}:\left(u,j,t\right)\notin H_{\mathrm{pre}}^{t}\cup H_{\tau ,k}^{t}\right\},$$

The training filter is constructed from the frozen bank and preceding events in the selected client’s task support. Validation and test use the common deterministic stage-valid sampler defined in Section 3. The positive and negative candidates are encoded symmetrically from pre-event states and candidate-specific contexts; labels and future events do not enter either context.

The total client loss is

(26) $$L_{k}=L_{k}^{\mathrm{task}}+\lambda_{p}{∥\Omega_{k}-\Omega^{\left(r\right)}∥}_{2}^{2}+\lambda_{a}L_{k}^{\mathrm{anchor}},$$

where $\Omega^{\left(r\right)}$ is the server prompt at round *r* and $L_{k}^{\mathrm{anchor}}$ aligns local prototypes with server-side temporal anchors. The proximal term follows the same motivation as FedProx in heterogeneous federated optimization, but it is applied only to prompt parameters [37].

### 4.4. Reliability-Weighted Aggregation and Training Procedure

Aggregation converts local temporal evidence into a shared prompt update. A simple mean gives the same influence to balanced support and to an update formed from one outcome. FedTIP instead computes each round-wise client weight solely from support size, label coverage, and update magnitude.

After local training, each active client sends $M_{k}^{\left(r\right)}$. Let $p_{k,c}=n_{k,c}/n_{k}$ for every task label c∈Y, with $p_{k,c}=0$ when client *k* has no example of class *c*. The label-coverage factor is   

(27) $$b_{k}=1-\frac{1}{2}\sum_{c\in Y}\left|p_{k,c}-\frac{1}{\left|Y\right|}\right|.$$

It equals one for a balanced local support distribution and decreases as the support concentrates on fewer outcomes. At round *r*, the update-norm threshold is determined from the active-client updates as

(28) $$\gamma^{\left(r\right)}={\mathrm{median}}_{k\in A_{\tau }}{∥\Delta \Omega_{k}^{\left(r\right)}∥}_{2}.$$

The reliability factor and normalized aggregation weight are

(29) $$r_{k}=b_{k}\min\left(1,\frac{\gamma^{\left(r\right)}}{∥\Delta \Omega_{k}^{\left(r\right)}{∥}_{2}+\epsilon }\right),$$

(30) $$w_{k}=\frac{n_{k}r_{k}}{\sum_{j\in A_{\tau }}n_{j}r_{j}},$$

Here, $\gamma^{\left(r\right)}$ is the median active-client update norm before reliability clipping and $\epsilon =10^{-8}$ prevents division by zero. The factor $n_{k}$ accounts for support size; $b_{k}$ accounts for label coverage; and the clipped norm factor limits unusually large updates. The global prompt is updated by   

(31) $$\Omega^{\left(r+1\right)}=\Omega^{\left(r\right)}+\eta_{s}\sum_{k\in A_{\tau }}w_{k}\Delta \Omega_{k}.$$

The server step $\eta_{s}$ scales the reliability-weighted prompt update, whereas β remains specific to anchor momentum. A single-class or single-outcome client therefore retains a nonzero weight, but an equally sized balanced client receives greater influence.

Algorithm 1 summarizes the procedure. The chronological split is created once, the temporal encoder is pre-trained centrally, and each downstream task federates only prompt-side adaptation over client-local support events.
**Algorithm 1** FedTIP Federated Temporal Interaction Prompting**Require:** 
Chronological events E, client partition, local downstream support sets, rounds *R***Ensure:** 
Global prompt adapter Ω and anchor prototypes $\bar{Q}$  1:Split E into pre-training, supervised-pool, validation, and test segments.  2:Pre-train temporal encoder $f_{\Theta_{0}}$ on historical link prediction; build $B_{0}$ and freeze $\Theta_{0}$.  3:Initialize prompt adapter $\Omega^{\left(0\right)}$ and anchor prototypes separately on the server.  4:**for** each downstream task **do**  5:    Select participating clients and construct their local support events from the task manifest.  6:    Deploy frozen bank parameters and metadata once to participating clients.  7:    **for** round r=0,…,R−1 **do**  8:        Server broadcasts $\Omega^{\left(r\right)}$ and anchor prototypes ${\bar{Q}}^{\left(r\right)}$.  9:        **for** client *k* in active clients **do**10:            Build event contexts from frozen-bank features and preceding local support events.11:            Sample negatives from client-visible $N_{k,t}^{\mathrm{tr}}\left(u\right)$ for link prediction.12:            Optimize $\Omega_{k}$ with task loss, proximal regularization, and prototype anchoring.13:            Upload the FedTIP message $M_{k}^{\left(r\right)}$.14:        **end for**15:        Server computes reliability weights $w_{k}$, updates anchors, and obtains $\Omega^{\left(r+1\right)}$.16:    **end for**17:    Select the configuration on the common server-side validation segment and report the chronological test score.18:**end for**

## 5. Experiments

The experiments evaluate predictive performance, access model effects, and cumulative uplink payload under the system model above. Federated methods share the same split manifest, client visibility, active-client set, and support budget. A matched-backbone comparison separates pooled prompt adaptation, direct FedAvg, and FedTIP; ablation and sensitivity analyses examine the reported components and partition conditions.

### 5.1. Experimental Setup

#### 5.1.1. Datasets and Temporal Protocol

The data protocol starts from raw temporal event tables and materializes a split manifest before training. Wikipedia and Reddit are user–content interaction graphs with binary dynamic user-state labels; MOOC is a student–course interaction graph with a binary dropout-related state label. Table 1 summarizes graph scale, feature dimensions, label imbalance, and six mutually exclusive chronological partitions. “NN-excl.” denotes downstream training-candidate events removed because they touch sampled inductive nodes, while “Ind. test” is a flagged subset of the test stage. Wikipedia and Reddit use 172-dimensional event features; MOOC uses 4-dimensional standardized event features.

The split is chronological and encoded in the manifest, following the temporal visibility convention used by TGAT, TGN, and subsequent temporal graph benchmarks [3,4,7]. The first 70% of each event sequence forms centralized pre-training, and the following 10% forms pre-training validation. Within the final 20%, the first 10% (approximately 2% of the complete stream) is the downstream training-candidate window, the next 10% is global downstream validation, and the remaining 80% is global testing. Sampling typed inductive users and items removes training-candidate events that touch those nodes, leaving supervised pools of 1810 (1.149%), 11,097 (1.650%), and 4699 (1.141%) events for Wikipedia, Reddit, and MOOC. The corresponding excluded counts are 1340, 2352, and 3536. The six manifest outcomes in Table 1 are disjoint and exhaustive for each raw event stream.

The main user-iid manifests use seed 2026 and assign each user to exactly one of ten clients before task generation. This gives 822–823, 1000, and 704–705 users per client on Wikipedia, Reddit, and MOOC. The corresponding client supervised pools contain 102–257, 996–1261, and 357–568 events. Each of the 100 generated tasks then samples exactly 30 supervised temporal interactions from five active clients, following the low-resource temporal prompt protocol of DyGPrompt [22]. The exact 30-event total defines the matched low-supervision comparison and equals 0.0190%, 0.00446%, and 0.00729% of the three raw streams. Uniform allocation together with the forced task-level positive yields 5–7 events per active client. The realized tasks contain 1.04, 1.02, and 1.32 positive state labels on average, respectively, and all 100 tasks per dataset contain both labels. On average, 1.04, 1.02, and 1.25 clients per task contain both labels. A client forms prototypes for its observed label set, and the global server-side scorer computes AUC-ROC. The 100 tasks are generated by repeated sampling from the shared supervised pool.

The sensitivity profile examines client coverage and partition skew on Wikipedia as a representative temporal interaction dataset. It varies the number of active supervised clients from two to ten and sets the total supervised temporal-event support to 6A for *A* active clients. The user-iid profile allocates this support approximately evenly across active clients. The label-skew profile keeps the total support fixed, but biases state-label composition across clients, so some clients observe mostly one label type. The activity-skew profile also keeps the same total support but makes client activity uneven, so some active clients hold more supervised temporal events than others. All client ownership files, task samples, validation segments, and test segments are materialized before model training and held fixed throughout evaluation.

#### 5.1.2. Baselines

Section 2 gives the baseline taxonomy. In the experiments, FedSage+, FedGCN, and FedGNN receive pre-downstream history-aggregated graph views; Feddy, STFL, FedDGL, and FedLink use their temporal or dynamic adaptation mechanisms. Each implementation is constrained to the same client-local downstream support and chronological visibility. FedLink provides the closest temporal federated comparator for link prediction.

The matched access model comparison uses TGAT [3] and TGN [4] with DyGPrompt [22]. For each backbone, the centralized row pools support events, the direct FedAvg row averages the same client-trained prompt parameters, and the FedTIP row uses the proposed adapter and aggregation under the same client visibility [8].

All federated comparisons share the chronological split, support budget, active-client set, and support ownership. A pre-trained baseline uses the same historical segment as FedTIP; other baselines start from the graph state or temporal features available before downstream supervision. Link prediction uses the historically common binary protocol of one negative per positive [7]. Negative destinations are generated by the same deterministic stage-valid rule and task/split seed for every method. The candidate pool contains destinations observed in pre-training history. The same_time rule excludes the positive destination and every true destination paired with the source at that timestamp; a stricter diagnostic mode additionally excludes every historical source–destination pair. Materializing both modes over 3000 support events per dataset gives zero positive collisions, zero same-time true-edge collisions, and zero sampling failures; the stricter mode also gives zero historical-pair collisions. Validation and test use the global chronological segments at the common server-side scorer.

#### 5.1.3. Evaluation Metrics

The primary metric is AUC-ROC for the three task families. Each table entry is the mean and standard deviation over 100 generated task identifiers, with link prediction reported separately on transductive and inductive subsets. Communication is the cumulative float32 uplink per downstream task under the same active-client profile.

#### 5.1.4. Implementation Details

Unless otherwise stated, FedTIP uses a TGN-style temporal memory encoder as the frozen bank; TGAT provides the matched alternative backbone. During downstream adaptation, each active client optimizes only the prompt-side parameters with Adam, a learning rate of $10^{-3}$, dropout of 0.1, and weight decay of $10^{-5}$; the temporal encoder remains frozen. Federation runs for R=20 rounds with E=5 local epochs per round. Each local epoch uses the complete client support as one batch, corresponding to 5–7 events under the main task manifests. The server step is $\eta_{s}=1.0$. Every similarity-softmax loss parameterized by τ in Section 4 uses τ=0.1.

The reliability rule sets $\gamma^{\left(r\right)}$ to the median active-client update norm in round *r* and uses $\epsilon =10^{-8}$ for numerical stability. The main mechanism coefficients are $\lambda_{p}=10^{-2}$, $\lambda_{a}=10^{-1}$, and β=0.1. They are selected by one-at-a-time validation sweeps over $\lambda_{p},\lambda_{a}\in \left\{0,10^{-4},10^{-3},10^{-2},10^{-1},1\right\}$ and β∈{0,0.01,0.05,0.1,0.5,0.9,1}, holding the other two coefficients and all fixed implementation settings at their main values. The experimental protocol fixes seed 2026, ten total clients, five active clients, 30 support events, 100 generated tasks, a 1:1 link-positive/negative ratio, deterministic negative-sampling seeds, and float32 communication accounting. Chronological split manifests, user-level ownership assignments, task-support allocations, client-indexed validation/test attribution views, and materialized support-stage negative selections instantiate these choices for all three datasets.

### 5.2. Main Results

Table 2 reports mean ± standard deviation over the 100 generated few-shot tasks. Federated graph baselines appear above federated dynamic-graph baselines; FedTIP uses the TGN-style frozen encoder. Bold and underlined values mark the highest and second-highest mean in each column.

As shown in Table 2, FedTIP has the highest reported mean in all nine cells. Relative to the strongest non-FedTIP mean in each column, the observed differences are +1.83, +3.51, and +2.26 points for node classification; +10.39, +12.19, and +3.31 for transductive link prediction; and +9.45, +14.69, and +4.06 for inductive link prediction. FedLink is the nearest non-FedTIP mean in most cells. The node-classification columns have smaller mean differences and larger across-task standard deviations than the link-prediction columns.

### 5.3. Access Model Comparison

Table 3 holds the prompt family and backbone fixed while changing downstream access and aggregation. Bold and underlined values mark the highest and second-highest means.

Table 3 shows that, averaged over the nine cells, direct FedAvg is 5.92 points below centralized TGAT-DyGPrompt and 6.08 points below centralized TGN-DyGPrompt. The corresponding task-family differences are 5.63, 6.28, and 5.87 points for TGAT and 6.19, 5.91, and 6.14 points for TGN (node classification, transductive link prediction, and inductive link prediction).

Relative to direct FedAvg, the FedTIP row is higher by 4.30 points with TGAT and 4.66 points with TGN on average. The task-family differences are 4.30, 4.44, and 4.17 points for TGAT and 4.50, 4.72, and 4.75 for TGN. The remaining mean difference from the centralized row is 1.62 points for TGAT and 1.42 points for TGN.

Table 3 also reveals a marked backbone difference on inductive link prediction. The TGAT means on Wikipedia (52.94) and MOOC (53.08) are close to chance, whereas TGN reaches 92.63 and 69.42 under centralized adaptation and 90.81 and 68.28 with FedTIP. The inductive subset contains nodes excluded from the supervised pool, making backbone choice especially consequential when query-time representations must be formed for unseen nodes from the same 30-event adaptation support.

### 5.4. Communication Efficiency

We measure client-to-server float32 payload. Let $P_{m}$ be the number of float32 values uploaded by method *m* over all active clients and rounds of one downstream task. The measured payload is

(32) $${\mathrm{MiB}}_{\mathrm{up}}\left(m\right)=\frac{4\cdot P_{m}}{1024^{2}},$$

**Figure 3:**
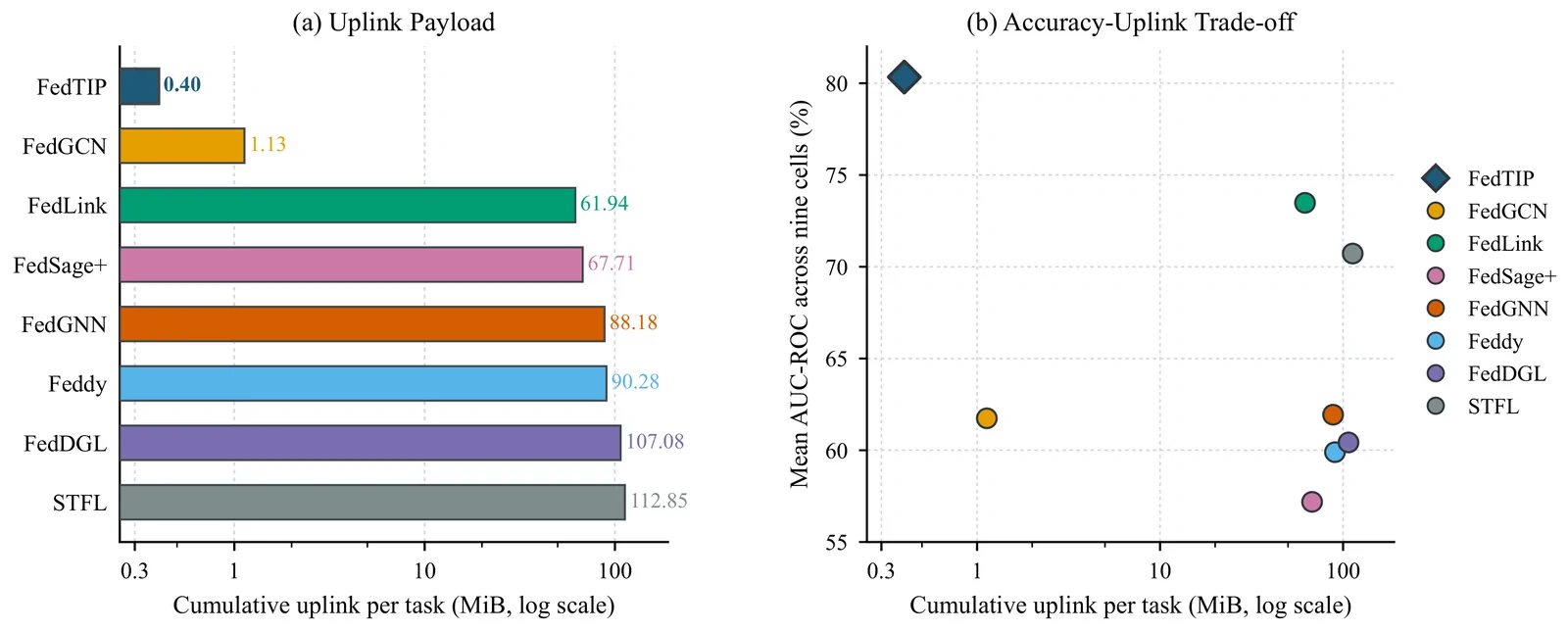
Communication efficiency under measured cumulative float32 uplink. (**a**) Uplink per downstream task, sorted by payload on a logarithmic axis. (**b**) Mean AUC-ROC across the nine task–dataset cells versus the same uplink.

The factor four is the number of bytes per float32 value. Method-specific uploaded updates and summaries are counted under the same rule. Figure 3 reports this measured cumulative uplink, while the subsequent equations characterize initialization and recurrent downlink.

Figure 3a reports 0.40 MiB for FedTIP, 1.13 MiB for FedGCN, and 61.94–112.85 MiB for the other baselines. Within the measured uplink boundary, these values give reductions of 64.6% relative to FedGCN and 99.4–99.6% relative to the remaining methods. FedGCN is the next-lowest uplink method, and its mean AUC-ROC is below FedTIP in each cell of Table 2.

Figure 3b uses the same measured uplink values and places FedTIP at the lowest-uplink and highest-mean position among the evaluated same-access methods. The cell-wise results in Table 2 support this aggregate position: FedTIP has the highest mean in every task–dataset cell.

Downlink has two distinct components. FedTIP deploys the frozen temporal bank once to each newly provisioned client; subsequent rounds broadcast only the global adapter and server-aggregated anchor prototypes. Let $N_{\mathrm{init}}$ be the number of clients receiving the bank, $A_{\tau ,r}$ the number of active clients in round *r* of task τ, and $R_{\tau }$ the number of rounds. Its downlink is therefore

(33) $$D_{\mathrm{FedTIP}}^{↓}\left(T\right)=N_{\mathrm{init}}\left|B_{0}\right|+\sum_{\tau =1}^{T}\sum_{r=1}^{R_{\tau }}A_{\tau ,r}\left(\left|\Omega \right|+\sum_{c}\left|{\bar{q}}_{c}\right|\right).$$

The first term is a reusable initialization cost, whereas the second contains only prompt-side state. FedSage+ [24], FedGCN [25], and FedGNN [23] repeatedly distribute graph model state and, where required, neighborhood or embedding information. Feddy [26] and STFL [27] synchronize dynamic or temporal–spatial model state; FedDGL [28] and FedLink [29] additionally exchange personalized, distillation, or temporal-embedding state. Writing $S_{m}$ for the recurrent server state of baseline *m* and $D_{m,\mathrm{aux}}^{↓}$ for its initialization and method-specific auxiliary transfers gives

(34) $$D_{m}^{↓}\left(T\right)=\sum_{\tau =1}^{T}\sum_{r=1}^{R_{m,\tau }}A_{\tau ,r}\left|S_{m}\right|+D_{m,\mathrm{aux}}^{↓}.$$

For FedTIP, the recurrent downlink is bounded by one adapter and one global-anchor set per active client and remains on the same sub-MiB parameter scale as its 0.40 MiB measured uplink. For baselines that exchange dense model state in both directions, the measured 1.13–112.85 MiB uplinks provide first-order estimates of recurrent model-state downlink under the same client and round schedule, with feature, embedding, and protocol transfers adding method-specific payload. Figure 3 provides the common measured-uplink comparison, and the equations above characterize the corresponding downlink structure. With a cached client cohort, the average FedTIP initialization term decreases as $N_{\mathrm{init}}\left|B_{0}\right|/T$, while recurrent full-state broadcasts remain proportional to the number of rounds. If $d_{m}^{↓}>d_{\mathrm{FedTIP}}^{↓}$ denotes the corresponding per-task recurrent costs, the symbolic amortization point is $T_{m}^{⋆}=\lceil N_{\mathrm{init}}|B_{0}\left|/\left(d_{m}^{↓}-d_{\mathrm{FedTIP}}^{↓}\right)\right\rceil$.

### 5.5. Ablation Study

Table 4 reports absolute AUC-ROC values for the full model, five single-component variants, and a joint prototype–aggregation variant. Each value first averages the 100 generated tasks within a dataset and then assigns equal weight to Wikipedia, Reddit, and MOOC. A cross denotes a removed component, whereas a blank entry denotes that the component is retained.

The variants preserve the main protocol and change only the stated mechanism. Removing TKB keeps the encoder architecture and downstream interface but omits historical self-supervised pre-training before the encoder is frozen. Removing ICP replaces the interaction-conditioned prompt with a static shared prompt. Removing PA sets $\lambda_{a}=0$ and suppresses global-anchor exchange and updates while retaining the local class prototypes required by node classification. Removing RWA sets $r_{k}=1$, which gives support-weighted FedAvg, $w_{k}=n_{k}/\sum_{j}n_{j}$, and class-anchor weights $a_{k,c}=n_{k,c}$. Removing FTN uses unfiltered destination sampling in both pre-training and downstream adaptation while still excluding the current positive destination. Variant 6 removes PA and RWA together and keeps TKB, ICP, FTN, and all remaining settings fixed.

As shown in Table 4, removing TKB decreases NC, T-LP, and I-LP by 5.11, 7.99, and 8.18 points relative to full FedTIP, the largest losses in the table. Removing ICP decreases the three task-family means by 3.18, 4.88, and 5.49 points. These patterns connect historical temporal knowledge and event-conditioned prompting to adaptation from sparse supervision. FTN is more task-specific: its removal decreases NC by 0.78 points but T-LP and I-LP by 5.54 and 5.73 points, respectively, consistent with negative destinations entering the link objective directly.

Table 4 further shows that removing PA decreases the three means by 2.52, 4.42, and 4.58 points, while replacing RWA with support-weighted FedAvg decreases them by 2.86, 4.12, and 4.40 points. Their joint removal produces larger losses than either single removal—3.50, 5.32, and 6.05 points—but smaller losses than the sum of the two single-component effects. To quantify this pattern, for task family τ we define

(35) $$I_{\mathrm{PA},\mathrm{RWA}}^{\tau }=\Delta_{\mathrm{PA}+\mathrm{RWA}}^{\tau }-\Delta_{\mathrm{PA}}^{\tau }-\Delta_{\mathrm{RWA}}^{\tau },$$

where $\Delta_{X}^{\tau }=M_{\mathrm{full}}^{\tau }-M_{-X}^{\tau }$. The resulting interaction contrasts are −1.88, −3.22, and −2.93 for NC, T-LP, and I-LP. The negative contrasts describe sub-additive overlap between PA and RWA, consistent with both mechanisms acting through the server-side prototype and client update aggregation path.

### 5.6. Core Hyperparameter Sensitivity

**Figure 4:**
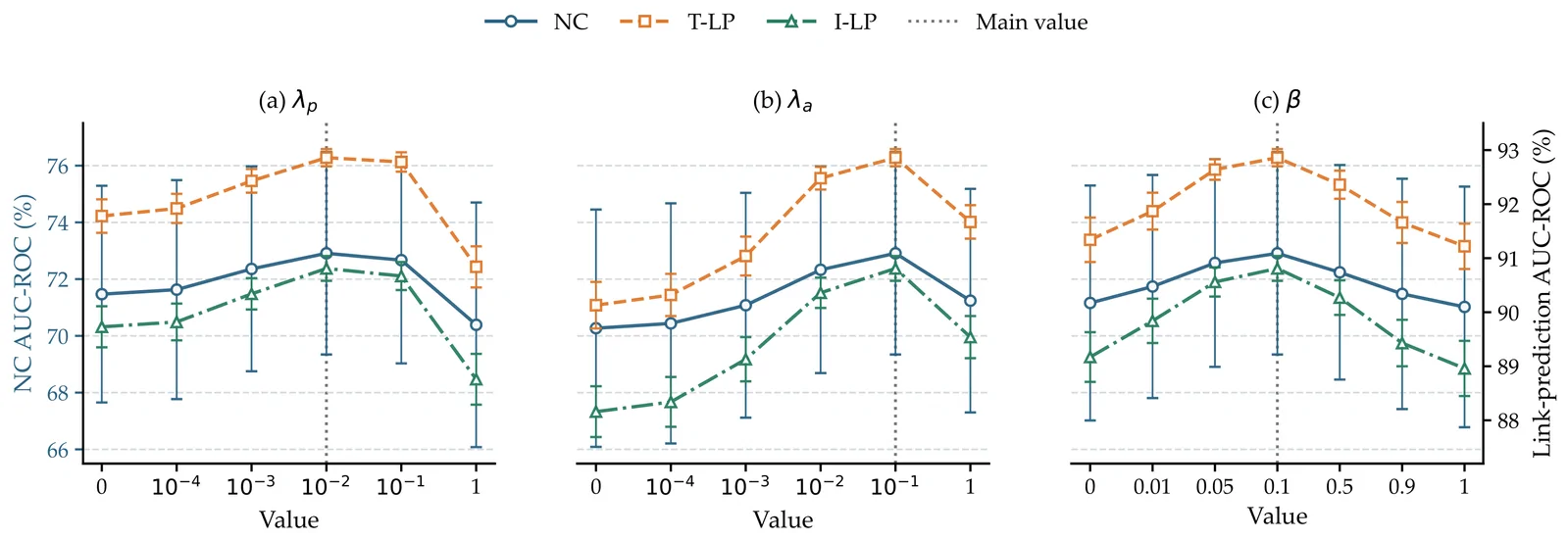
One-at-a-time sensitivity of core FedTIP hyperparameters on Wikipedia under the main low-supervision setting. Curves show mean AUC-ROC (%), error bars show the standard deviation over 100 generated tasks, and vertical dotted lines mark the main configuration. NC uses the left axis; T-LP and I-LP use the right axis.

Figure 4 examines the three coefficients that directly regulate downstream adaptation. Each one-at-a-time sweep uses the same 100 Wikipedia task identifiers under the main low-supervision setting, with the other two coefficients and all protocol settings fixed at their main values. The error bars quantify task-level dispersion across these controlled comparisons.

Figure 4 shows that the selected $\lambda_{p}=10^{-2}$ improves NC, T-LP, and I-LP by 1.44, 1.08, and 1.08 points over $\lambda_{p}=0$, and by 2.52, 2.02, and 2.05 points over $\lambda_{p}=1$. This interior optimum balances client-specific adaptation against proximal control: removing the term permits greater local drift, whereas an excessive coefficient suppresses useful prompt updates. Similarly, $\lambda_{a}=10^{-1}$ improves the three tasks by 2.64, 2.73, and 2.65 points over no prototype anchoring and by 1.67, 1.19, and 1.27 points over $\lambda_{a}=1$, indicating that moderate class-level alignment is useful without forcing heterogeneous clients too tightly toward the global anchors.

For anchor momentum, β=0.1 exceeds β=0 by 1.75, 1.52, and 1.64 points and β=1 by 1.89, 1.64, and 1.85 points on NC, T-LP, and I-LP. The result favors strong historical smoothing with a modest current-round update: β=0 completely preserves the previous anchor and prevents incorporation of current client evidence, whereas β=1 follows the current-round aggregate without historical carry-over. Across all three sweeps and task families, the selected values attain both the highest mean and the smallest observed task-level standard deviation.

### 5.7. Client Coverage, Support Scaling, and Partition Skew

**Figure 5:**
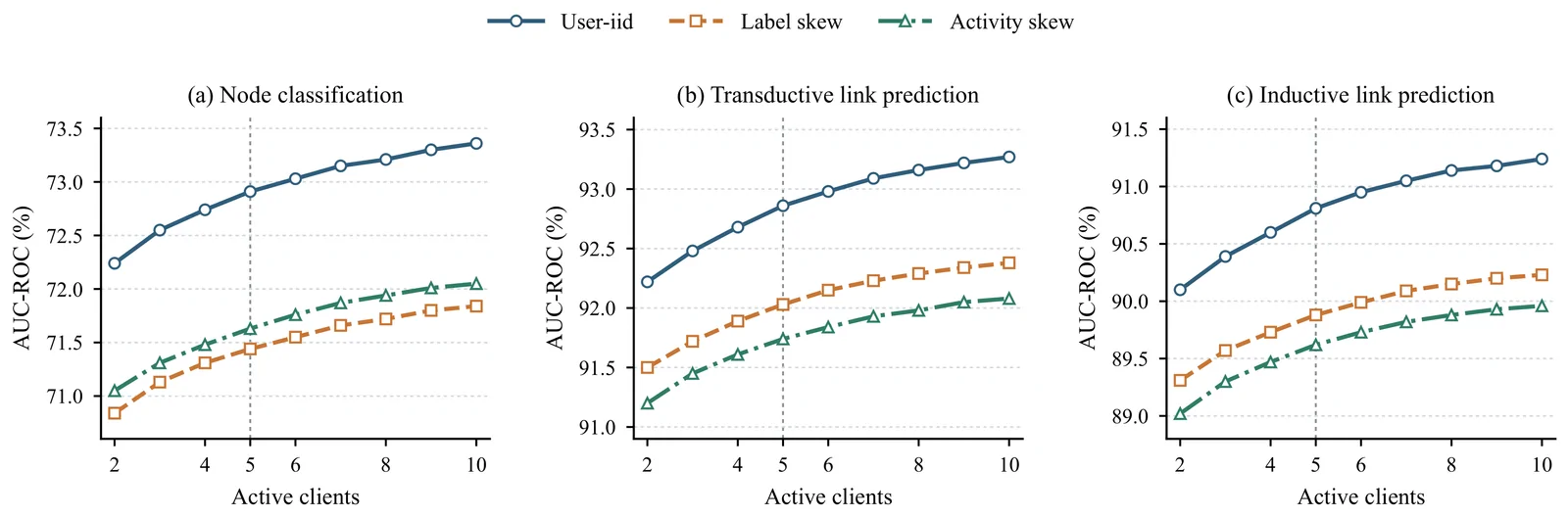
Wikipedia client coverage, support scaling, and partition-skew analysis; all three profiles use 6A support events at active-client count *A*.

Figure 5 varies the active-client count *A* from two to ten on Wikipedia and sets total support to 6A events. The horizontal trend therefore measures the joint effect of broader client coverage and increased supervision. At fixed *A*, user-iid, label-skew, and activity-skew use the same support count, isolating the effect of the partition profile.

As shown in Figure 5, under user-iid partitioning, increasing *A* from two to ten raises AUC-ROC from 72.24 to 73.36 for node classification, 92.22 to 93.27 for transductive link prediction, and 90.10 to 91.24 for inductive link prediction. The five-client points (72.91, 92.86, and 90.81) match the Wikipedia FedTIP row in Table 2. Beyond five clients, each curve gains less than 0.5 points.

Figure 5 further shows that, at five active clients, label skew is 1.47, 0.83, and 0.93 points below user-iid, while activity skew is 1.28, 1.12, and 1.19 points below. At ten clients, the respective gaps are 1.52, 0.89, and 1.01 points for label skew and 1.31, 1.19, and 1.28 for activity skew.

### 5.8. Limitations and Scope

The study has three principal scope boundaries. First, historical pre-training and downstream validation are centralized; the locality scope covers downstream support events. Second, the cross-method quantitative bars cover cumulative float32 uplink under complete participation of the selected clients. Downlink is characterized from each method’s exchanged objects and communication cadence: FedTIP combines one-time frozen-bank deployment with recurrent adapter-and-anchor broadcasts, whereas the compared full-state methods repeatedly distribute substantially larger model or auxiliary state. Protocol headers and retransmissions remain outside this parameter-payload analysis. Third, the structured uplink exposes prompt updates, prototype summaries, counts, and reliability fields to the coordinator. Section 3.5 characterizes this message-level threat surface, derives the small-count prototype sensitivity, and establishes secure-summation and sensitivity-calibrated interfaces for the aggregation rule.

## 6. Conclusions

FedTIP formulates the temporal prompt adapter as the federated trainable object and uses coverage-aware joint prompt–prototype aggregation to govern both prompt deltas and class-anchor geometry. On Wikipedia, Reddit, and MOOC, it has the highest reported mean AUC-ROC in the nine same-access cells and a measured cumulative uplink of 0.40 MiB per task. The message-level analysis further characterizes prototype exposure and shows how FedTIP’s additive aggregation interface composes with secure summation and sensitivity-calibrated prototype release. Together, these results establish the practical value of prompt-level federation for chronologically ordered few-shot support.

## Figures and Tables

**Table 1:** Dataset statistics and chronological event partitions.

Dataset	Raw Event Statistics							Chronological Manifest Counts						
	Events	Users	Items	Feat. Dim	Classes	Pos. Labels	Pos. Rate	Pre-Train	Pre-Val	Sup. Pool	Down-Val	Test	NN-Excl.	Ind. Test
Wikipedia	157,474	8227	1000	172	2	217	0.001378	110,232	15,747	1810	3150	25,195	1340	11,333
Reddit	672,447	10,000	984	172	2	366	0.000544	470,713	67,244	11,097	13,449	107,592	2352	17,344
MOOC	411,749	7047	97	4	2	4066	0.009875	288,224	41,176	4699	8235	65,879	3536	30,817

**Table 2:** Mean AUC-ROC (%) for node classification and transductive and inductive link prediction; standard deviations are over 100 generated tasks.

Method	Node Classification AUC			Transductive LP AUC			Inductive LP AUC		
	Wiki.	Reddit	MOOC	Wiki.	Reddit	MOOC	Wiki.	Reddit	MOOC
FedSage+	61.84 ± 8.91	54.72 ± 7.36	56.31 ± 5.84	58.76 ± 1.64	62.10 ± 2.27	52.88 ± 1.31	56.21 ± 1.87	59.43 ± 1.52	52.46 ± 2.36
FedGCN	66.33 ± 6.74	58.96 ± 6.29	61.74 ± 4.97	63.58 ± 2.18	69.72 ± 1.96	55.41 ± 1.42	59.84 ± 2.43	64.87 ± 1.74	55.23 ± 2.05
FedGNN	54.69 ± 9.82	62.83 ± 5.67	50.94 ± 5.21	72.15 ± 0.38	66.08 ± 1.45	54.07 ± 0.93	73.46 ± 0.41	68.35 ± 1.16	54.88 ± 1.37
Feddy	63.48 ± 6.51	56.29 ± 7.24	64.02 ± 4.88	61.76 ± 2.84	68.91 ± 2.16	51.62 ± 1.03	58.74 ± 2.61	62.39 ± 2.35	51.87 ± 1.78
STFL	70.16 ± 5.18	67.58 ± 6.93	58.74 ± 5.64	76.88 ± 1.26	82.41 ± 1.13	63.92 ± 2.04	75.31 ± 1.52	79.02 ± 1.36	62.46 ± 2.21
FedDGL	56.42 ± 8.37	64.37 ± 5.95	62.76 ± 5.16	68.53 ± 0.91	59.84 ± 2.73	56.73 ± 0.68	61.08 ± 1.19	57.96 ± 2.48	56.15 ± 2.67
FedLink	71.08 ± 4.76	68.91 ± 5.34	65.38 ± 4.42	82.47 ± 1.58	83.12 ± 1.87	65.33 ± 3.16	81.36 ± 1.43	79.48 ± 2.11	64.22 ± 3.08
FedTIP	**72.91** ± 3.57	**72.42** ± 3.26	**67.64** ± 4.03	**92.86** ± 0.16	**95.31** ± 0.09	**68.64** ± 0.82	**90.81** ± 0.23	**94.17** ± 0.11	**68.28** ± 0.71

**Table 3:** Mean AUC-ROC (%) for centralized prompting, direct FedAvg, and FedTIP with matched TGAT or TGN backbones.

Method	Node Classification			Transductive LP			Inductive LP		
	Wiki.	Reddit	MOOC	Wiki.	Reddit	MOOC	Wiki.	Reddit	MOOC
TGAT-DyGPrompt (centralized)	**82.64** ± 6.61	73.06 ± 6.32	**77.41** ± 5.21	70.21 ± 0.27	91.02 ± 0.14	54.36 ± 1.04	52.94 ± 0.32	75.71 ± 0.24	53.08 ± 0.95
TGAT-DyGPrompt (FedAvg)	76.48 ± 8.63	67.91 ± 8.41	71.84 ± 7.34	63.72 ± 1.74	84.61 ± 1.29	48.43 ± 2.64	46.94 ± 1.68	69.37 ± 1.91	47.82 ± 2.49
TGAT-FedTIP	80.73 ± 6.58	72.09 ± 6.72	76.31 ± 5.21	68.42 ± 0.24	89.28 ± 0.13	52.37 ± 1.08	51.09 ± 0.28	73.84 ± 0.21	51.72 ± 0.94
TGN-DyGPrompt (centralized)	74.12 ± 3.62	**74.39** ± 3.25	69.54 ± 4.08	**94.01** ± 0.19	**96.46** ± 0.11	**69.91** ± 0.87	**92.63** ± 0.25	**95.38** ± 0.13	**69.42** ± 0.79
TGN-DyGPrompt (FedAvg)	68.36 ± 5.94	68.14 ± 5.21	62.97 ± 6.42	88.17 ± 1.38	90.42 ± 1.12	64.06 ± 2.31	86.11 ± 1.46	89.43 ± 1.03	63.48 ± 2.18
TGN-FedTIP	72.91 ± 3.57	72.42 ± 3.26	67.64 ± 4.03	92.86 ± 0.16	95.31 ± 0.09	68.64 ± 0.82	90.81 ± 0.23	94.17 ± 0.11	68.28 ± 0.71

**Table 4:** Ablation study on FedTIP design components.

Methods	TKB	ICP	PA	RWA	FTN	Mean AUC-ROC		
						NC	T-LP	I-LP
Variant 1	×					65.88	77.61	76.24
Variant 2		×				67.81	80.72	78.93
Variant 3			×			68.47	81.18	79.84
Variant 4				×		68.13	81.48	80.02
Variant 5					×	70.21	80.06	78.69
Variant 6			×	×		67.49	80.28	78.37
FedTIP						70.99	85.60	84.42

## Data Availability

Wikipedia, Reddit, and MOOC are public benchmark datasets distributed by their original providers. The reproducibility materials include chronological split manifests, user-level client assignments, task-support allocations, client-indexed validation/test attribution views, materialized support-stage negative selections, and measured communication and prototype-support summaries for the three datasets. These materials are available from the corresponding author upon reasonable request.
